# Is I-FABP not only a marker for the detection abdominal injury but also of hemorrhagic shock in severely injured trauma patients?

**DOI:** 10.1186/s13017-019-0267-9

**Published:** 2019-11-21

**Authors:** Maika Voth, Thomas Lustenberger, Borna Relja, Ingo Marzi

**Affiliations:** Department of Trauma, Hand and Reconstructive Surgery, University Hospital, Goethe University Frankfurt, Theodor-Stern-Kai 7, D-60590 Frankfurt, Main, Germany

**Keywords:** I-FABP, Hemorrhagic shock, Emergency, Abdominal trauma, Biomarker

## Abstract

**Background:**

Hemorrhagic shock can lead to intestinal damage with subsequent hyperinflammation and multiple organ dysfunction syndrome (MODS). The intestinal fatty acid-binding protein (I-FABP) is solely expressed in the intestine and is released extracellulary after tissue damage. This study evaluates the validity of I-FABP as an early biomarker to detect hemorrhagic shock and abdominal injury.

**Patients and methods:**

Severely injured patients with an Injury Severity Score (ISS) ≥ 16 points and an age ≥ 18 years, admitted from January 2010 to December 2016, were included. Overall, 26 patients retrospectively presented with hemorrhagic shock to the emergency room (ER): 8 patients without abdominal injury (“HS noAbd”) and 18 patients with abdominal injury (“HS Abd”). Furthermore, 16 severely injured patients without hemorrhagic shock and without abdominal injury (“noHS noAbd”) were retrospectively selected as controls. Plasma I-FABP levels were measured at admission to the ER and up to 3 days posttraumatic (d1-d3).

**Results:**

Median I-FABP levels were significantly higher in the “HS Abd” group compared with the “HS noAbd” group (28,637.0 pg/ml [IQR = 6372.4–55,550.0] vs. 7292.3 pg/ml [IQR = 1282.5–11,159.5], *p* < 0.05). Furthermore, I-FABP levels of both hemorrhagic shock groups were significantly higher compared with the “noHS noAbd” group (844.4 pg/ml [IQR = 530.0–1432.9], *p* < 0.05). The time course of I-FABP levels showed a peak on the day of admission with a subsequent decline in the post-traumatic course. Furthermore, significant correlations between I-FABP levels and clinical parameters of hemorrhagic shock, such as hemoglobin, lactate value, systolic blood pressure (SBP), and shock index, were found.

The optimal cut-off level of I-FABP for detection of hemorrhagic shock was 1761.9 pg/ml with a sensitivity of 85% and a specificity of 81%.

**Conclusion:**

This study confirmed our previous observation that I-FABP might be used as a suitable early biomarker for the detection of abdominal injuries in general. In addition, I-FABP may also be a useful and a promising parameter in the diagnosis of hemorrhagic shock, because of reflecting low intestinal perfusion.

## Introduction

In severely injured trauma patients, traumatic brain injury and uncontrolled bleeding remain the main causes for early mortality within the first 24 h after trauma [1].

Hemorrhagic shock results in a circulatory dysfunction causing decreased tissue oxygenation and an accumulation of oxygen debt [2].

In experimental models, tissue hypoperfusion has extensively been reported as a crucial pathophysiological event leading to tissue hypoxia and thereby organ failure [3, 4]. Furthermore, hemorrhagic shock leads to a decrease of the fraction of perfused intestinal villi and thereby to an increase of the risk of villous ischemia [5]. Thereby it leads to intestinal damage with disruption of tight junction complexes and subsequent failure of the gut barrier [6–8].

This resulted in a translocation of luminal bacteria and leads to hyperinflammatory response and secondary complications like systemic inflammatory distress syndrome (SIRS), sepsis and multi-organ dysfunction syndrome (MODS) [9–13], which are main causes for late mortality of severely traumatized patients [14, 15].

This intestinal damage can be verified using the intestinal fatty acid-binding protein (I-FABP). FABPs are small intracellularly or within the plasma membrane localized proteins and are released into the extracellular space in their soluble extracellularly form early after cell or tissue damage [16]. Therefore, FABPs are used as plasma and urine markers for tissue-specific injuries [17]. The measurement of FABPs levels can be performed within hours by ELISA [18]. For heart-type FABP (H-FABP), a bedside test (qualitative rapid immunochemical point-of care-tests, POCT) is available, providing results within 15 min [19, 20].

Among the nine organ-specific isoforms of FABP, the I-FABP is solely present in enterocytes of the entire small intestine and partly in the colon and appears rapidly in the circulation after intestinal epithelial cell damage [21, 22]. I-FABP has been studied as a biomarker for the diagnosis of necrotizing enterocolitis [23], the detection of mesenteric ischemia [24, 25] and strangulated mechanical small bowel obstruction [26]. In our own studies, we previously demonstrated that I-FABP is a significant marker for abdominal trauma [27–29]. Recently, a correlation between intestinal hypoperfusion and plasma I-FABP values was found [8].

For the present study, we hypothesized that I-FABP, as a marker for intestinal damage, could not only be a novel plasma marker in the early phase after trauma for the detection of abdominal injury but also for hemorrhagic shock, which leads to intestinal hypoperfusion and damage.

## Patients and methods

### Study design

This pilot study was performed at the University Hospital Frankfurt of the Goethe University with an Institutional Ethics Committee approval (312/10, in accordance with the Declaration of Helsinki and reported following the Strengthening the Reporting of OBservational studies in Epidemiology, STROBE guidelines) [30]. Written informed consent was obtained from all enrolled subjects or their nominated legally authorized representatives on behalf of the participants in accordance with the ethical standards.

### Patients

Severely injured patients with an Injury Severity Score (ISS) ≥ 16 points [31] and ≥ 18 years of age were included at admission to the emergency room (ER), in which sequential blood measurement over 3 days could be achieved. The study period was January 2010 to December 2016. Further inclusion criteria consisted of a history of acute blunt or penetrating trauma. Patients with burns, concomitant acute myocardial infarction, chronic diseases, and lethal injuries were excluded.

During the study period 26 patients retrospectively presented with hemorrhagic shock according to our definition outlined below and according to our inclusion and exclusion criteria: 8 patients without an abdominal injury (“HS noAbd”) and 18 patients with an abdominal injury (“HS Abd”).

Furthermore, 16 severely injured patients (ISS ≥ 25) were coincidentally selected as control patients. These patients did not have any abdominal injury nor did they present in hemorrhagic shock (“noHS noAbd”).

### Data collection

Upon arrival to the ER vital parameters of all patients were recorded. Each injury was assigned an AIS score by a trained physician at hospital discharge and the ISS was calculated.

Abdominal injury was defined as an injury of the kidney, liver, spleen, pancreas, bladder, ureter and urethra, abdominal blood vessels, and intestine with an AIS abdomen ≥ 3 points. The patient’s characteristics were obtained from the patient’s digital files.

For the present study, hemorrhagic shock was defined using the following criteria:

- Positive shock index (SI) (≥ 1) prehospital or in the ER and
- Hemoglobin (Hb) < 10 g/dl in the ER and
- Lactate value ≥ 4 mmol/l in the ER and
- The need of a massive transfusion (≥ 10 packed red blood cells (PRBC) within the first 24 h.

### Sample collection

Blood samples were obtained at admission to the ER (d0) and daily for 3 days (d1–d3) following trauma. Blood samples were collected in prechilled ethylenediaminetetraacetid acid tubes (BD Vacutainer, Bectom Dickinson Diagnostics, Aalst, Belgium) and kept on ice. Blood was centrifuged at 2000×*g* for 15 min at 4 °C. The supernatant was stored at − 80 °C until the batch sample analysis. Blinded specimens were used for duplicate measurement of I-FABP levels. I-FABP levels were determined by the laboratory of the Department of Trauma, Hand and Reconstructive Surgery at the Hospital of the Goethe University Frankfurt using a highly specific commercially available ELISA (Hycult Biotechnology, Uden, The Netherlands) according to manufacturer’s instructions.

Blood sampling for the measurement of I-FABP was started in the year 2010. For the purpose of this study and as previously described, 26 patients with hemorrhagic shock were retrospectively identified and the I-FABP assays from the banked blood were performed in the year 2017. Furthermore, 16 severely injured patients (ISS ≥ 25) without hemorrhagic shock and without abdominal injury were selected coincidentally as controls and I-FABP assays were run likewise.

### Data analysis

The Kolmogoroff-Smirnoff-Lilleford’s test showed that the plasma concentrations of I-FABP were not Gaussian distributed. Median I-FABP levels of the three groups (“noHS noAbd” vs. “HS noAbd” vs. “HS Abd”) were compared using the Kruskal-Wallis test. In order to deal post hoc on non-parametric data, the Mann-Whitney *U*-test was applied and the Bonferroni adjustment of the *p* value to correct for multiple comparisons was performed. Data are presented as the median and interquartile range (IQR) unless stated otherwise. A *p* value of < 0.05 was considered statistically significant.

Spearman’s correlation coefficients were calculated to determine correlations between I-FABP levels and other variables.

Sensitivity, specificity, positive predictive value (PPV), and negative predictive value (NPV) were calculated and receiver operator characteristic curves (ROC) were generated to analyze the optimal cut-off level of I-FABP.

Bias 7.0 (Epsilon Verlag GbR 1989–2009, Germany) and GraphPad Prism 3.02 (GraphPad Software Inc. San Diego, CA) were used to perform the statistical analysis and computations.

## Results

### Patient’s characteristics

Forty-two patients were enrolled in this study. Table 1 depicts the patients’ demographic and injury characteristics. No statistically significant differences were found comparing the three groups regarding age, gender, ISS, hospital and intensive care unit (ICU) length of stay, and mortality. In the “noHS noAbd” group, a significantly higher AIS score of the head was noticed compared with the “HS Abd” group; however, no significant difference was found comparing both hemorrhagic shock groups. Furthermore, as per definition, the AIS abdomen score was significantly higher in the “HS Abd” group compared with both other patient groups (*p* < 0.05).

**Table 1:** Summary of the patient’s demographic and injury characteristics and the in-hospital outcome

	noHS noAbd (*n* = 16)	HS noAbd (*n* = 8)	HS Abd (*n* = 18)	*p* value all groups	*p* value (HS noAbd vs. HS Abd.)
Age	48 (30–56)	44 (27.8–48.5)	44.5 (38–62.8)	0.76	0.64
Sex (male, *n*, %)	12 (75%)	7 (87.5%)	15 (83.3%)	0.72	1.0
ISS	34.5 (32–41)	34 (33.8–35.8)	42 (36.5–50)	0.13	0.19
AIS					
Head	4 (3–5)	0 (0–4)	0 (0–3)	0.001	0.57
Face	0 (0–3)	1 (0–2)	0 (0–1)	0.46	0.36
Chest	3 (3–4)	3 (2–3)	4 (2–5)	0.25	0.20
Abdominal	0 (0–0)	0 (0–0)	4 (3–4)	< 0.0001	< 0.0001
Extremity	3 (2–4)	5 (3–5)	3 (2–4)	0.16	0.18
Injury pattern (*n*, blunt: penetrating)	16: 0	6: 2	16:2	0.14	0.56
ICU length of stay (days)	16 (11.5–23.8)	17.5 (4.5–31.8)	21.5 (10.5–29.0)	0.68	0.74
Hospital length of stay (days)	22 (14.8–35.8)	23.5 (11.0–43.3)	28.0 (18.3–38.8)	0.86	0.78
In-hospital Mortality (*n*, %)	2 (12.5%)	2 (33.3%)	4 (22.2%)	0.69	1.0

Values are reported as median (interquartile range, IQR) and as percentages

*AIS*, Abbreviated Injury Scale Score; *HS Abd*, hemorrhagic shock and abdominal injury; *HS noAbd*, hemorrhagic shock without abdominal injury; *ICU*, intensive care unit; *ISS*, Injury Severity Score; *noHS noAbd*, no hemorrhagic shock and no abdominal injury

Table 2 outlines the physiologic characteristics of the three patient groups.

**Table 2:** Physiologic characteristics of the patient groups

Physiologic characteristics	noHS noAbd (*n* = 16)	HS noAbd (*n* = 8)	HS Abd (*n* = 18)	*p* value all groups	*p* value (HS noAbd vs. HS Abd.)
I-FABP (pg/ml, ER)	844.4 (530.0–1432.9)	7292.3 (1282.5–11,159.5)	28,637.0 (6372.4–55,550.0)	< 0.0001	0.02
SBP (mm Hg, pre-hospital)	130 (95–151)	115 (88–129)	70 (68–90)	0.0004	0.02
SBP (mm Hg, ER)	105 (86–120)	100 (78–113)	71 (61–106)	0.11	0.40
heart rate (*n*/min, prehospital)	91 (80–110)	120 (110–121)	116 (100–130)	0.05	0.73
heart rate (*n*/min, ER)	101 (90–110)	115 (101–123)	120 (88–132)	0.29	0.98
shock index (prehospital)	0.7 (0.5–1.0)	1.0 (0.9–1.4)	1.4 (1.3–1.8)	0.0002	0.05
shock index (ER)	1.0 (0.8–1.3)	1.2 (1.0–1.4)	1.4 (1.1–2.0)	0.09	0.39
Fluid transfusion (ml, pre-hospital)	1000 (650–1000)	1250 (500–1750)	1500 (1000–1500)	0.28	0.88
Fluid transfusion (ml, ER)	1000 (775–2250)	3000 (1937.5–6375)	2000 (1500–2500)	0.02	0.08
PRBC transfusion within 24 h (Units)	2 (1–5.5)	11.5 (10.8–22.5)	19 (13–27.8)	< 0.0001	0.18
PRBC transfusion total (Units)	6 (3.5–12.5)	19.5 (15.5–32.3)	25.5 (18–37.5)	< 0.0001	0.44
FFP transfusion within 24 h (Units)	0 (0–3)	10 (5.8–17)	15.5 (11.3–19)	< 0.0001	0.19
FFP transfusion total (Units)	0 (9–3)	10 (5.8–19)	17.5 (11.5–21.3	< 0.0001	0.16
Hemoglobin (g/dl, ER)	11.9 (11.4–13.5)	6.6 (5.2–8.0)	7.1 (5.7–8.4)	< 0.0001	0.49
PTT (s, ER)	28 (24.5–30)	48 (38–51.5)	38 (29–45)	0.002	0.1
INR (ER)	1.1 (1–1.3)	1.3 (1.2–1.8)	1.3 (1.1–1.5)	0.06	0.32
Fibrinogen (mg/dl, ER)	190.5 (153–242.5)	142 (116.3–176)	186.5 (134–227.5)	0.24	0.23
PLT count (× 10^3^/μl, ER)	186.5 (155.8–256.8)	94 (51.3–137.8)	178.5 (113.3–206.8)	0.03	0.03
Hemostatic therapy (done, n, %)	8 (50%)	8 (100%)	18 (100%)	0.0003	1.0
Leukocyte count (U/nl)	10.8 (9.5–13.5)	9.9 (8.8–11)	8.6 (6.7–10.8)	0.14	0.47
Lactate (mmol/l, ER)	2.4 (1.9–3.9)	7.4 (6.4–10.6)	8.5 (6.7–9.2)	< 0.0001	0.98
pH value	7.3 (7.2–7.4)	7.4 (7.1–7.4)	7.2 (7.1–7.2)	0.03	0.26
Base deficit (mmol/l, ER)	− 5.1 (−7.1–− 3.2)	− 7.2 (− 11.8–− 1.9)	− 10.1 (− 11.7–− 6.3)	0.11	0.45
Temperature (°C, ER)	36 (35.3–36.5)	37 (35.9–37.5)	36.1 (35.4–36.6)	0.28	0.13

Values are reported as median (interquartile range, IQR)

*ER*, emergency room; *FFP*, fresh frozen plasma; *HS Abd*, hemorrhagic shock with abdominal injury; *HS noAbd*, hemorrhagic shock without abdominal injury; *I-FABP*, intestinal fatty acid-binding protein; *INR*, international normalized ratio; *noHS noAbd*, no hemorrhagic shock and no abdominal injury; *PLT*, platelets; *PRBC*, packed red blood cells; *PTT*, partial thromboplastin time; *SBP*, systolic blood pressure

Statistically significant differences were found comparing the prehospital systolic blood pressure (SBP), the prehospital SI, the fluid resuscitation, transfusion of PRBC within 24 h and overall, transfusion of fresh frozen plasma (FFP) within 24 h and overall, hemoglobin value, partial thromboplastin time (PTT), platelet count, lactate, pH value, and need of hemostatic therapy (*p* < 0.05) between the three patient groups.

Comparing the “HS noAbd” group with the “HS Abd” group, a significant difference in the prehospital SBP and in the platelet counts was found.

### I-FABP level as a marker of severe abdominal injury and hemorrhagic shock

**Fig. 1:**
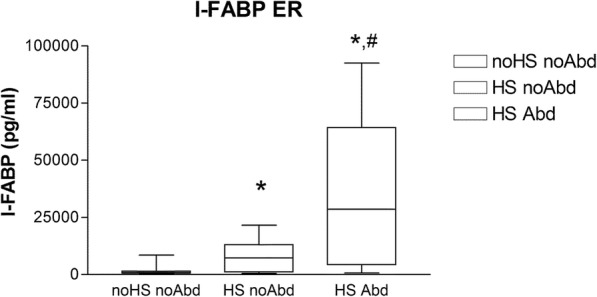
I-FABP levels on admission to the emergency room. Median (interquartile range, IQR) intestinal fatty acid-binding protein (I-FABP) values in the three study groups based on the presence or absence of hemorrhagic shock and abdominal injury on admission to the emergency room. noHS noAbd (*n* = 16); HS noAbd (*n* = 8); HS Abd (*n* = 18). *, *p* < 0.05 HS noAbd vs. noHs noAbd; HS Abd vs. noHS noAbd. #, *p* < 0.05 HS Abd vs. HS noAbd

The median concentrations of I-FABP at admission to the ER were significantly higher in the “HS Abd” group (28,637.0 pg/ml [IQR = 6372.4–55,550.0]) and in the “HS noAbd” group (7292.3 pg/ml [IQR = 1282.5–11,159.5]) compared with the “noHS noAbd” group (844.4 pg/ml [IQR = 530.0–1432.9], *p* < 0.05) (Fig. 1). Furthermore, the median I-FABP level was significantly higher in the “HS Abd” group compared with the “HS noAbd” group (Fig. 1, *p* < 0.05).

### I-FABP in the 3-day posttraumatic course

**Fig. 2:**
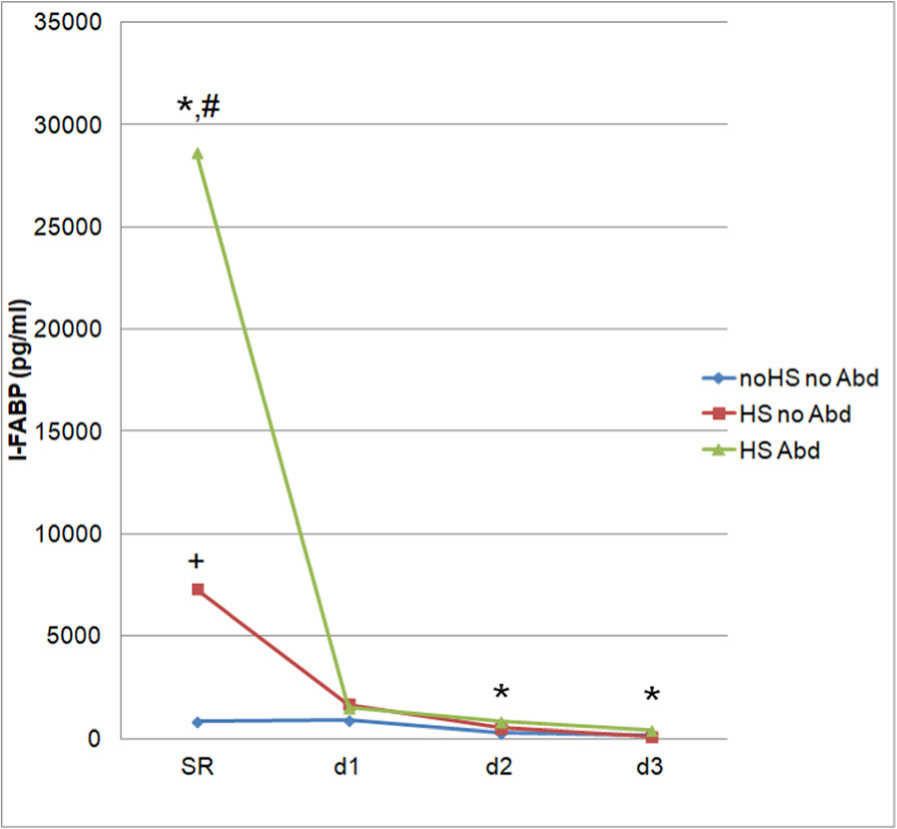
Time course of I-FABP levels. Time course of median intestinal fatty acid-binding protein (I-FABP) levels (pg/ml) of all three patient groups based on the presence or absence of hemorrhagic shock and abdominal injury. ER, emergency room; HS Abd, hemorrhagic shock with abdominal injury; HS noAbd, hemorrhagic shock without abdominal injury; noHS noAbd, without hemorrhagic shock and without abdominal injury. *, *p* < 0.05 HS Abd. vs. noHS noAbd; #, *p* < 0.05 HS Abd. vs. HS noAbd; +, *p* < 0.05 HS noAbd vs. noHS noAbd

Figure 2 demonstrates the 3-day time-course of I-FABP for the three patient groups. In the ER, significantly higher I-FABP levels were found in “HS Abd” group compared with the other two patient groups. Likewise, I-FABP levels were statistically significantly higher in “HS noAbd” patients compared with the “noHS noAbd” group.

Following the first peak at admission (ER), I-FABP levels decreased in all three patient groups over the observed time course. On day 2 and 3, significantly higher I-FABP levels were noticed in the “HS Abd” patient group compared with the “noHS noAbd” group (Fig. 2, *p* < 0.05).

### I-FABP correlates with clinical parameters for hemorrhagic shock

**Fig. 3:**
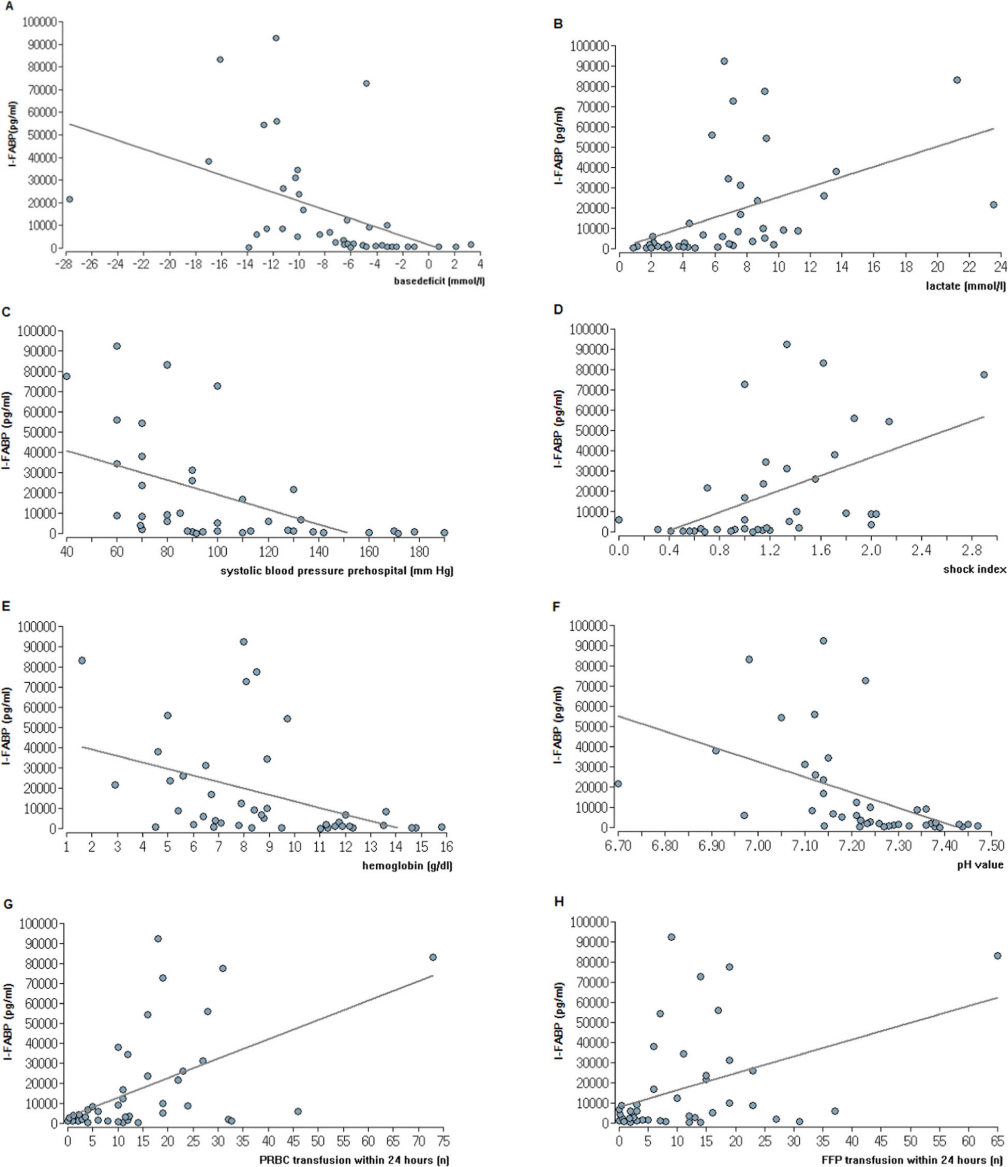
Correlations of I-FABP levels with clinical parameters of shock. Spearman rank correlations of I-FABP levels (*n* = 26) and different clinical parameters of shock on the day of admission (ER). **a** Base deficit, *ρ* = − 0.62, *p* < 0.0001. **b** Lactate value, *ρ* = 0.72, *p* < 0.0001. **c** Systolic blood pressure prehospital, *ρ* = − 0.65, *p* < 0.0001. **d** Shock index prehospital, *ρ* = 0.62, *p* < 0.0001. **e** Hemoglobin, *ρ* = − 0.54, *p* = 0.0002. **f** pH value, *ρ* = − 0.73, *p* < 0.0001. **g** Packed red blood cells transfusion (PRBC) within the first 24 h, *ρ* = 0.61, *p* < 0.0001. **h** Fresh frozen plasma transfusion (FFP) within the first 24 h, *ρ* = 0.49, *p* = 0.001

The I-FABP levels at admission to the ER significantly correlated with the following clinical hemorrhagic shock parameters: base deficit (Fig. 3a), lactate value (Fig. 3b), prehospital and ER SBP (Fig. 3c), prehospital and ER SI (Fig. 3d), Hb value (Fig. 3e), pH value (Fig. 3f) and the amount of PRBC and FFP units transfused within the first 24 h and overall (Fig. 3g+h, respectively).

Furthermore, statistically significant correlations between I-FABP levels at admission to the ER and leukocyte counts, international normalized ratio (INR), and PTT levels were found.

There were no statistically significant correlations between I-FABP levels and fluid transfusion, platelet counts, fibrinogen, temperature, and ISS.

### ROC analysis for optimal cut-off level of I-FABP in hemorrhagic shock

**Fig. 4:**
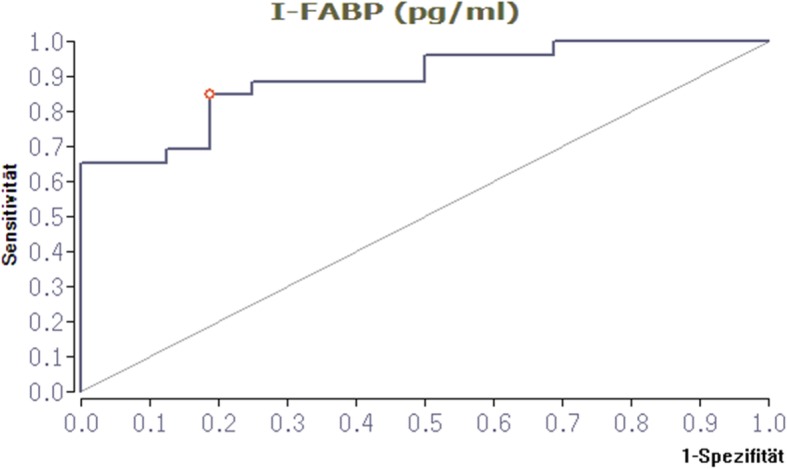
Receiver operating curve of I-FABP for detection of hemorrhagic shock. Receiver operating characteristic curve showing the optimal cut-off for I-FABP levels (1761.9 pg/ml) in predicting the presence or the absence of hemorrhagic shock with 85% sensitivity and 81% specificity. I-FABP, intestinal fatty acid-binding protein

Receiver operating characteristic curve analysis shows an optimal cut-off level of I-FABP of 1761.9 pg/ml for detecting hemorrhagic shock, with 85% sensitivity and 81% specificity. The area under the curve (AUC) is = 0.89 (Fig. 4).

The positive predictive value (PPV) and the negative predictive value (NPV) for I-FABP for detection of hemorrhagic shock were 88% and 76.5%, respectively.

## Discussion

One of the main causes for early mortality in severely traumatized patients is massive bleeding in almost 50% [32]. Hemorrhagic shock leads to tissue hypoperfusion [3, 4] and intestinal damage [6, 8] consequently resulting in a translocation of bacteria, inflammatory response, and subsequently MODS [9, 12, 13, 33]. These resulting secondary complications like SIRS, sepsis, and MODS [9–13] are main causes for late mortality of severely traumatized patients [14, 15].

The present study investigated the association between abdominal injury and I-FABP, as well as the association between hemorrhagic shock and I-FABP, as a marker for intestinal damage because of a reduced circulation and tissue hypoperfusion.

I-FABP is a primary marker for abdominal injury. In patients presenting with hemorrhagic shock to the ER, the presence of an abdominal injury significantly affected the I-FABP levels in this study. These results confirm our previously published results, showing that I-FABP is significantly increased in patients with abdominal injuries compared with patients without abdominal injuries [27, 29]. In this respect, we have demonstrated earlier that I-FABP levels are increased in patients with a perforation or rupture of the small or large intestine [28]. The delayed diagnosis of an intestinal injury increases the risk of sepsis, MODS, acute respiratory distress syndrome, and mortality [34–37]. In fact, 3 of 4 patients with a delayed diagnosis had severe complications during the further clinical course [28].

The severity of trauma reveals a significant relationship with the I-FABP levels [38, 39]. In our own data, we have similarly reported a significant correlation between I-FABP levels and the overall severity of trauma, defined by using the ISS [29].

In the present study, however, the ISS was not statistically significant different between the different groups. This excluded a relevant impact of the overall injury severity on the measured I-FABP levels. Additionally, we have not observed significant differences concerning the specific patient characteristics between the three patient groups in this current study.

The new finding of the data presented is that patients with hemorrhagic shock exhibited a significantly higher I-FABP level at admission to the ER as compared with those patients without hypovolemia. Most importantly, this difference was independent from the presence or absence of an abdominal injury. We could calculate an optimal cut-off level for I-FABP for detection of a hemorrhagic shock of 1761.9 pg/ml with a sensitivity of 85% and a specificity of 81. This shows the potential usefulness of I-FABP as an early marker for hemorrhagic shock or—intestinal hypoperfusion by that. The detection of intestinal hypoperfusion and thereby resulting intestinal damage is still an unsolved problem in the clinical setting, due to the lack of direct access and of specific markers [40–42]. From all, I-FABP is the most promising biomarker for the detection of intestinal hypoperfusion [41].

To the best of our knowledge, this is the first study presenting a profound evidence for a significant correlation between I-FABP levels and hemorrhagic shock in trauma patients. In previously published studies, correlations between I-FABP and hypoperfusion of the intestine were found [8]. Furthermore, other studies showed increased I-FABP levels in patients with low Hb values, low mean arterial pressure (MAP), and elevated SI, and were likewise related to the severity of trauma [29, 38, 39]. However, in particular due to compensatory mechanisms of the human body, the two clinical parameters, low blood pressure and SI, are unreliable parameters in determining the presence of hemorrhagic shock. It has been shown that none of these parameters is adequately sensitive or specific to detect early hemorrhage [43]. Serum markers like lactate or base deficit determine tissue hypoperfusion and global tissue acidosis, respectively, and are the most commonly used serum markers for hemorrhagic shock [44–48]. These two parameters likewise correlated with I-FABP in the present study. Additionally, correlations were found with routinely used and clinically relevant parameters such as the Hb value and SBP on admission, amount of PRBC and FFP transfused, SI, and pH value. Also, INR and PTT, both markers of coagulation disorders, significantly correlated with I-FABP levels on admission. Overall, our results suggest that I-FABP could serve as a novel marker for the detection of hemorrhagic shock.

The present analysis has several limitations, most importantly the limited number of patients enrolled. Future studies should involve larger cohorts of patients and controls to confirm our findings and to analyze sensitivity and specificity of I-FABP levels to detect hemorrhagic shock, abdominal injury, and injuries to specific abdominal organs in particular. In particular, it might be relevant to evaluate whether I-FABP is an independently early-detectable and sensitive marker of hemorrhagic shock. Such a marker would allow early improved or maybe even a monitoring of shock therapy.

Likewise, since we observed an early decrease of the initially elevated I-FABP levels to normal values, the usefulness of FABP assays in the clinical setting needs to be evaluated in a prospective setting.

Furthermore, no bedside-test for a rapid measurement of I-FABP currently exists for these situations. The I-FABP testing is performed using an ELISA test, taking 2 to 4 h for measurement and therefore limiting its clinical relevance in the acute setting up to now. Otherwise, the measurement of I-FABP by using ELISA is easy and the cost is only 13 Euros per patient. Introducing I-FABP on clinical routine would stimulate the development of a point of care approach, as it was already developed for H–FABP, with a bedside test with available results within 15 min for acute coronary syndrome or myocardial infarction [19].

## Conclusion

In conclusion, I-FABP levels not only identify patients with abdominal trauma but also allow the detection of hemorrhagic shock, most likely due to the hypoperfusion of the intestine in this situation. Thus, I-FABP is a useful and promising early marker for the detection of abdominal injury and even in the absence of an abdominal injury it is a marker of intestinal damage and hemorrhagic shock.

## Data Availability

The datasets during and/or analyzed during the current study available from the corresponding author on reasonable request.

## Abbreviations

AUC: Area under the curve
ER: Emergency Room
FFP: Fresh frozen plasma
Hb: Hemoglobin
H-FABP: Heart-type fatty acid binding protein
HS Abd: Patients with hemorrhagic shock and abdominal injury
HS noAbd: Patients with hemorrhagic shock and without abdominal injury
ICU: Intensive care unit
I-FABP: Intestinal fatty acid binding protein
INR: International normalized ratio
IQR: Interquartile range
ISS: Injury Severity Score
MAP: Mean arterial pressure
MODS: Multiple organ dysfunction syndrome
noHS noAbd: Patients without hemorrhagic shock and without abdominal injury
NPV: Negative predictive value
PPV: Positive predictive value
PRBC: Packed red blood cells
PTT: Partial thromboplastin time
ROC: Receiver operator characteristic curves
SBP: Systolic blood pressure
SI: Shock index
SIRS: Systemic inflammatory distress syndrome
